# Supplementary material for: Evaluation of an Online Version of the CFT 20-R in Third and Fourth Grade Children
Source: Children (Basel). 2022 Apr 4;9(4):512. doi: 10.3390/children9040512 (PMC9029809; doi:10.3390/children9040512)
Supplement: Supplementary file 1 [file children-09-00512-s001.zip › Supplementary Material children-1650115.pdf]

Supplementary Table S1. Item difficulties

Series

		Item difficulties		
item	correct answer	online (N = 4 100)	online (N = 220)	PP
1	d	.98	.98	1.00
2	b	.96	.96	.98
3	e	.99	.99	.96
4	***a***	***.28***	***.27***	***.90***
5	e	.92	.92	.89
6	b	.71	.74	.92
7	c	.74	.75	.89
8	c	.58	.62	.59
9	d	.59	.60	.76
10	***a***	***.20***	***.20***	***.60***
11	***b***	***.39***	***.41***	***.70***
12	***a***	***.32***	***.30***	***.73***
13	c	.40	.43	.28
14	d	.30	.37	.35
15	e	.28	.25	.21

Notes. ***Numbers in bold and italic*** show differences in item difficulties ≥ .20; PP = Paper-Pencil.

Classifications

		Item difficulties		
item	correct answer	online (N = 4 100)	online (N = 220)	PP
1	d	.99	1.00	.98
2	a	.79	.76	.88
3	b	.84	.88	.90
4	a	.73	.75	.78
5	e	.76	.75	.86
6	c	.55	.55	.63
7	***b***	***.58***	***.54***	***.77***
8	a	.39	.41	.47
9	c	.41	.45	.55
10	***e***	***.78***	***.76***	***.25***
11	c	.32	.35	.36
12	e	.40	.40	.45
13	d	.41	.38	.39
14	d	.36	.35	.24
15	b	.09	.10	.07

Notes. ***Numbers in bold and italic*** show differences in item difficulties ≥ .20; PP = Paper-Pencil.

Matrices

		Item difficulties		
item	correct answer	online (N = 4 100)	online (N = 220)	PP
1	b	.96	.96	1.00
2	c	.98	.98	1.00
3	b	.95	.96	.99
4	d	.96	.96	.97
5	***b***	***.41***	***.43***	***.71***
6	***a***	***.56***	***.60***	***.79***
7	***e***	***.57***	***.59***	***.90***
8	***d***	***.55***	***.58***	***.85***
9	***c***	***.34***	***.39***	***.60***
10	***a***	***.20***	***.21***	***.64***
11	e	.50	.52	.62
12	c	.41	.43	.38
13	***d***	***.59***	***.63***	***.35***
14	e	.33	.35	.16
15	a	.11	.08	.05

Notes. ***Numbers in bold and italic*** show differences in item difficulties ≥ .20; PP = Paper-Pencil.
